# Anti-Toxin Notes

**Published:** 1896-01

**Authors:** 


### ANTI-TOXIN NOTES.

CHICAGO, October 25th.—Dr. William H. Cook, of this city, who has spent forty years in the pursuit and practice of his profession, has prepared for distribution among physicians of the city, a pamphlet in which he denounces the use of the anti-toxin remedy for diphtheria as insanity, and scores the Commissioner of Health of Chicago for permitting the department physicians to use the serum. He says that horse serum produces blood-poisoning, and that doctors are fools to inject it into human blood. The doctor declares that some persons are so susceptible to the fad that it kills them, and says the "fad" will disappear in a year.

Diphtheria Statistics.—The action of the Health Boards in requesting that all cases of sore throat be promptly reported, will do much to swell the list of anti-toxin statistics.—*Med. Brief, St. Louis.*

Fitting Facts to Theories.—For a long time bacteriologists found it impossible to inoculate animals with typhoid fever. Then Cenarelli came to the rescue. He placed the animal to be operated on in a *polluted atmosphere,* injected his little germ, and secured a typical case. There is not much to be said about the *honesty* of such experiments.—*Med. Brief, St. Louis.*

Reasonable Suggestion.—If by tapping an old horse that has been filled with diphtheria bacilli doctors can get a cure for the disease, why shouldn't the doctors tap an old Kentucky Colonel and get a toxin that would knock the gold cure out of *sight?* The matter is respectfully referred to Dr. Seguin.—*The General Practitioner*, Vol. I, December, 1895, No. 12.

